# Advertising Specialties

**Published:** 1868-03-01

**Authors:** 


### *Advertising Specialties.*

Albeit in "goodlie companie," the Editor acknowledges being "sold" in a recent case. "For particulars see small bills," and the pages of a monthly contemporary in this city. An inch was given, an ell taken, whereupon our contemporary waxes mildly savage, and gives the takers the — *ell* with an aspirate. "Thereby hangs a tale," which we have not time, just now, to unfold; because to-day we descend from the tripod, throw physic for the nonce to the dogs, and, for a few days only, leave this great and wicked city, where the carcasses do so much abound which the eaglets, of the specialist variety alluded to, do scent afar off. We seek a breath of God's pure air, and on our return may launch an arrow at venereal and venal advertisers, not forgetting a quiver full thereof at sundry others who advertise their professional wares under the foxy captions "Medical Education," or "Medical Reform."

